# Pulmonary Hilar Tumor: An Unusual Presentation of Sclerosing Hemangioma

**DOI:** 10.1155/2016/8919012

**Published:** 2016-09-27

**Authors:** Jui-Hung Hung, Ching Hsueh, Chiung-Ying Liao, Shang-Yun Ho, Yuan-Chun Huang

**Affiliations:** Department of Medical Imaging, Changhua Christian Hospital, No. 135 Nanxiao St., Changhua 500, Taiwan

## Abstract

Pulmonary sclerosing hemangioma is an uncommon benign tumor of the lung; however, on rare occasions it can arise from the pulmonary hilar region. Herein, we report a 53-year-old female patient who presented with a round opacity in the right upper lung field on a radiograph. Chest computed tomography scanning revealed a 3.1 cm mass in the right pulmonary hilum. Thoracoscopic tumor excision was subsequently performed. On pathohistologic study, the tumor was well defined and composed of round stromal cells and surface cells arranged in a papillary, sclerotic, solid, and hemorrhagic pattern. In immunochemical study, the round cells were positive for thyroid transcription factor-1 (TTF-1) and epithelial membrane antigen (EMA) and negative for cytokeratin. The surface cells were positive for TTF-1, EMA, and cytokeratin. Therefore, a final diagnosis of sclerosing hemangioma was confirmed. In conclusion, pulmonary sclerosing hemangioma is uncommon and rare in the pulmonary hilar region. CT scanning is useful to determine its benignity, although imaging features are not specific for a definite differential diagnosis from other pulmonary tumors. Therefore, tissue diagnosis is usually necessary, and pulmonary sclerosing hemangioma should be listed in the differential diagnoses of pulmonary hilar tumors.

## 1. Introduction

Pulmonary sclerosing hemangioma is an uncommon benign tumor of the lung; however, on rare occasions it can arise from the pulmonary hilar region. The condition is sometimes referred to as “pneumocytoma,” because it is considered to be a pulmonary epithelial tumor, rather than a vascular tumor as the name implies [1]. Pulmonary sclerosing hemangioma is usually slow-growing and does not cause symptoms [2]. We describe herein a rare case of pulmonary hilar sclerosing hemangioma and present the relevant imaging and pathologic findings, along with a review of the literature. To our knowledge, this is the first documented case of pulmonary sclerosing hemangioma as an intrapulmonary hilar primary tumor.

## 2. Case Report

A 53-year-old female was referred to our hospital due to a round opacity in the right upper lung field on a radiograph. She denied any symptoms or a history of smoking. Family history was negative for relevant diseases. A hemogram was normal, and there were no increases in the concentrations of tumor markers.

**Figure 1:**
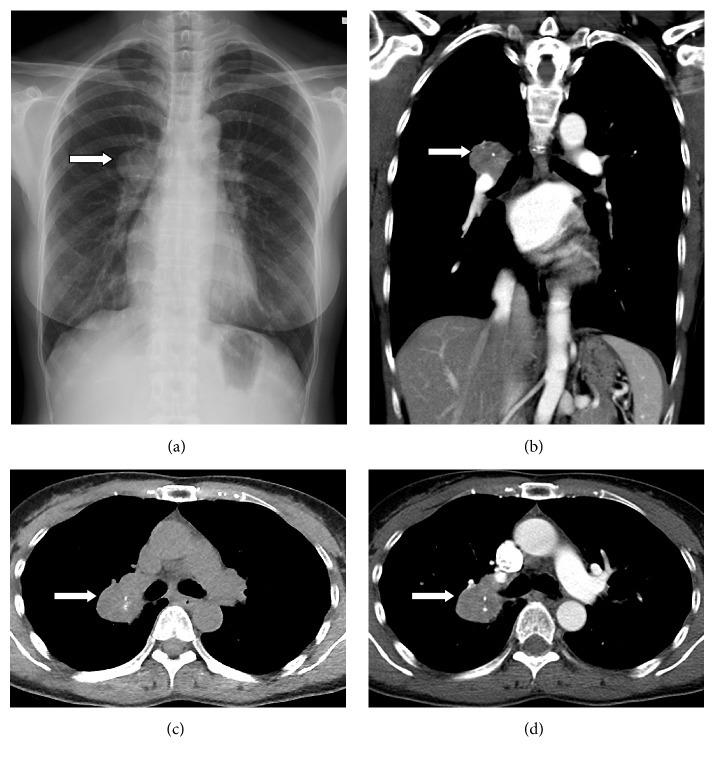
(a) Chest X-ray study detects a mass (white arrow) in the right pulmonary hilar region. (c) Precontrast CT scan reveals a well-defined right hilar mass with flecks of calcification and some low attenuation area. ((b) and (d)) Contrast enhanced CT scan shows a heterogeneous enhanced mass in the right pulmonary hilar region.

**Figure 2:**
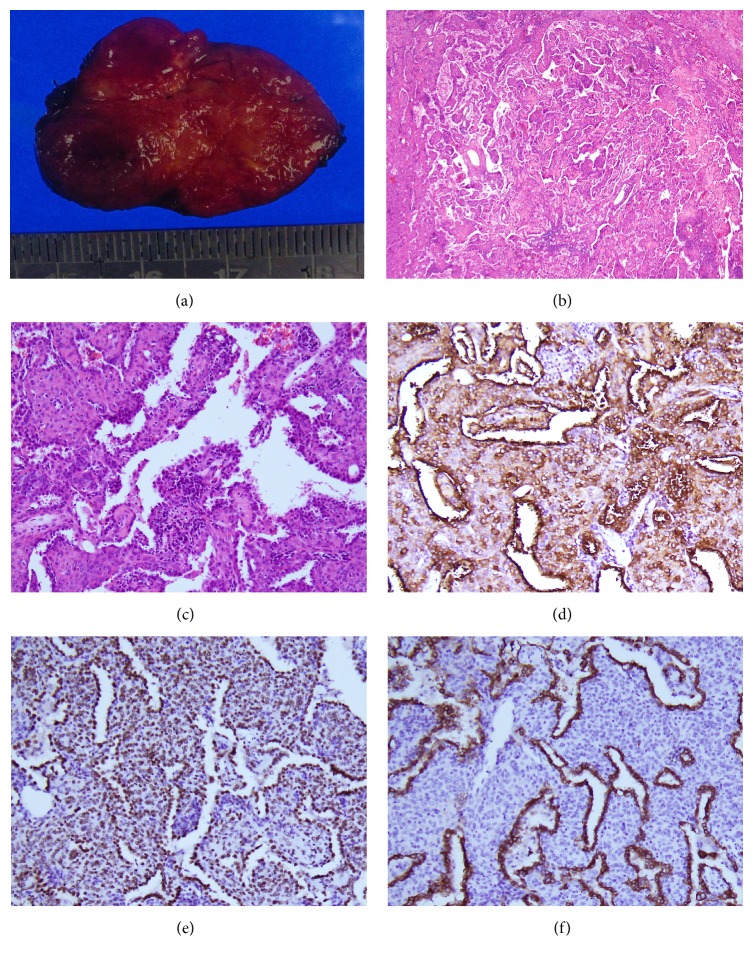
Histopathological analysis. (a) The gross specimen of the pulmonary hilar tumor is brownish and well circumscribed. (b) Microscopically, it shows a well-defined tumor composed of two cell types with round stromal cells and surface cells arranged in papillary, sclerotic, solid, and hemorrhagic pattern. Round cells are small with centrally located round to oval bland nuclei. Cuboidal surface cells are morphologically resembling type II pneumocyte. Lymphoplasmacytic infiltrate, xanthoma cells, hemosiderin, and calcification are seen (H&E stain, ×200). (c) In high power view, papillary configuration is revealed, formed by cuboidal surface cells and round stromal cells (H&E stain, ×400). (d) Photomicrographs show EMA and (e) TTF-1 are positive for both surface cells and stromal cells. (f) Cytokeratin is positive for surface cells and negative for stromal cells. Taken together, sclerosing hemangioma is diagnosed.

A chest radiograph demonstrated a mass in the right pulmonary hilar region (Figure 1(a)). Chest computed tomography (CT) scanning revealed a 3.1 cm mass in the right pulmonary hilum, between the right upper lobe and the right intermediate bronchus (Figure 1(b)). The mass was well circumscribed, with several scattered calcifications (Figure 1(c)). After intravenous iodinated contrast media injection, the mass showed heterogeneous enhancement (Figure 1(d)). There was no associated mediastinal lymph node enlargement. Bronchoscopy showed no endobronchial invasion. The patient underwent a CT-guided biopsy to investigate the mass, and pathologic examination revealed sclerosing hemangioma. The patient then underwent another chest CT scan six months later, which showed no significant change of the mass. Thoracoscopic tumor excision was subsequently performed, and the tumor appeared grossly brownish and firm (Figure 2(a)). On pathohistologic study, the tumor was well defined and composed of round stromal cells and surface cells arranged in a papillary, sclerotic, solid and hemorrhagic pattern. Cuboidal surface cells morphologically resembled type II pneumocytes (Figures 2(b) and 2(c)). Lymphoplasmacytic infiltrates, xanthoma cells, hemosiderin, and calcification were observed. In immunochemical study, the round cells were positive for thyroid transcription factor-1 (TTF-1) and epithelial membrane antigen (EMA) and negative for cytokeratin (Figures 2(d), 2(e), and 2(f)). The surface cells were positive for TTF-1, EMA, and cytokeratin. Therefore, a final diagnosis of sclerosing hemangioma was confirmed. The patient recovered well after the operation, and a follow-up chest CT scan showed no recurrence of the tumor.

## 3. Discussion

In a scenario of a mass in the central lung, we usually consider differential diagnoses including bronchogenic tumor, carcinoid tumor, lymphoma, granulomatous infection, or lymphadenopathy including a metastatic lymph node. It is difficult to obtain a definite diagnosis of primary pulmonary tumor based on imaging findings alone, although some image features may help to narrow down the list of differential diagnoses.

Pulmonary sclerosing hemangioma is an uncommon benign tumor of the lung, usually observed in Asian middle-aged women. This diagnosis was first proposed in 1956 by Liebow and Hubbell [1]. Most pulmonary sclerosing hemangiomas are solitary and are located in the peripheral zone of the lung, although multiple unilateral or bilateral lesions and central lesions have been reported. The tumor size ranges from 0.3 to 8 cm [3, 4]. The disease typically causes no symptoms and is usually found incidentally on a radiograph; however, patients may sometimes present with nonspecific symptoms such as a cough or hemoptysis [5].

On pathohistologic examination, the tumor presents a solid, papillary, hemangiomatous, and sclerotic pattern. At least three of these patterns are observed in the majority of patients. Two cell types can be found: cuboidal cells and round cells [5]. Tumor cells express EMA, whereas the round cell component lacks pancytokeratin expression. TTF-1 expression in both surface cells and round cells is strongly suggestive of primitive respiratory epithelial derivation [3, 6].

Typical presentations on CT scans include a well-delineated mass with good enhancement [7]. The tumor may contain high-, iso-, and low-attenuating areas, reflecting the angiomatous, sclerosing, and cystic components, respectively. Calcification may be present in 41% of patients with the disease [8]. Small tumors of less than 3 cm usually enhance well and homogeneously, while larger tumors may show inhomogeneous enhancement. The poorly enhanced area represents cystic components in the tumor [5]. Other imaging features include an air-meniscus sign similar to aspergilloma, a tail sign formed by a tail-like projection from the tumor, and a prominent pulmonary artery due to increased arterial demand [1, 8].

Atypical presentations of sclerosing hemangioma such as clustered pulmonary nodules with focal ground glass infiltration in the same lobe of the lung have been reported, which mimic pulmonary tuberculosis or other infiltrative lung diseases [9]. Although pulmonary sclerosing hemangiomas are generally considered to be benign lesions, regional lymph node metastasis has also been reported, although prognosis is not affected. Young male patients are prone to sclerosing hemangioma with nodal metastasis, and the tumor size is usually larger [2]. Pulmonary sclerosing hemangioma combined with other different tumors in the lung is extremely rare. Liu et al. reported a young female with sclerosing hemangioma combined with primary adenocarcinoma of the lung [10].

Surgical excision is curative without the need for additional treatment. For large sclerosing hemangioma, lymph node dissection may be needed due to possible lymph node metastasis [1]. Our patient finally received surgery due to a minor possibility of the tumor harboring malignancy, although it was observed to be of benign appearance on imaging study without interval change during the 6-month follow-up period. These imaging characteristics may suggest a benign process but are not specific enough to make a definite differentiation from other pulmonary tumors.

## 4. Conclusion

Pulmonary sclerosing hemangioma is uncommon and rare in the pulmonary hilar region. CT scanning is useful to determine its benignity, although imaging features are not specific for a definite differential diagnosis from other pulmonary tumors. Therefore, tissue diagnosis is usually necessary, and pulmonary sclerosing hemangioma should be listed in the differential diagnoses of pulmonary hilar tumors.
